# Potential approaches to the treatment of Ewing's sarcoma

**DOI:** 10.18632/oncotarget.12566

**Published:** 2016-10-11

**Authors:** Hongjiu Yu, Yonggui Ge, Lianying Guo, Lin Huang

**Affiliations:** ^1^ Department of Pathophysiology, Dalian Medical University, Dalian, Liaoning, P.R. China; ^2^ Department of VIP, The First Affiliated Hospital, Dalian Medical University, Dalian, Liaoning, P.R. China

**Keywords:** Ewing's sarcoma, targeted therapy, immunotherapy

## Abstract

Ewing’s sarcoma (ES) is a highly aggressive and metastatic tumor in children and young adults caused by a chromosomal fusion between the Ewing sarcoma breakpoint region 1 (*EWSR1*) gene and the transcription factor *FLI1* gene. ES is managed with standard treatments, including chemotherapy, surgery and radiation. Although the 5-year survival rate for primary ES has improved, the survival rate for ES patients with metastases or recurrence remains low. Several novel molecular targets in ES have recently been identified and investigated in preclinical and clinical settings, and targeting the function of receptor tyrosine kinases (RTKs), the fusion protein EWS-FLI1 and mTOR has shown promise. There has also been increasing interest in the immune responses of ES patients. Immunotherapies using T cells, NK cells, cancer vaccines and monoclonal antibodies have been considered for ES, especially for recurrent patients. Because understanding the pathogenesis of ES is extremely important for the development of novel treatments, this review focuses on the mechanisms and functions of targeted therapies and immunotherapies in ES. It is anticipated that integrating the knowledge obtained from basic research and translational and clinical studies will lead to the development of novel therapeutic strategies for the treatment of ES.

## INTRODUCTION

Ewing's sarcoma (ES) is an aggressive and highly metastatic malignancy predominantly afflicting young patients. The reciprocal chromosomal translocation t (11;22) (q24;q12) is found in 85% of these tumors, which leads to the fusion between the 5′ segment of the Ewing sarcoma breakpoint region 1 gene (*EWSR1*) on the chromosome 22 and the 3′ portion of Friend leukemia virus integration site 1 (*FLI1*) on the chromosome 11. In addition to FLI1, fusions between EWSR1 and other ETS family transcription factors, including ATF-1, ERG, and WT1, occur in ES. These translocations produce the chimeric proteins EWS-ETSs, which function as aberrant transcription factors, accounting for the tumorigenic potential of ES.

The standard care for patients suffering from ES is based on a multimodal therapy of surgical resection associated with local radiotherapy and chemotherapy [1–4]. This strategy has markedly improved the patient outcome; current 5-year survival rate for patients with localized ES has increased up to 70%. Nevertheless, the 5-year survival rate remains less than 20% for patients with metastatic or recurrent tumors [2, 3, 5]. Several clinical studies have indicated that the survival rate of ES patients receiving conventional multimodal therapy has reached a plateau phase [4, 6]. Therefore, novel therapies are urgently needed to improve the treatment; targeted therapies and immunotherapies seem most promising.

## TARGETED THERAPY

### Receptor tyrosine kinases (RTKs)

RTKs mediate key signaling pathways involved in cell proliferation, survival, migration and differentiation. Abnormal RTK signaling often leads to cell transformation, which is observed in a wide variety of malignancies. RTK has been targeted also in ES, although little abnormal expression of RTKs has been detected.

#### Insulin-like growth factor 1 receptor

**Figure 1:**
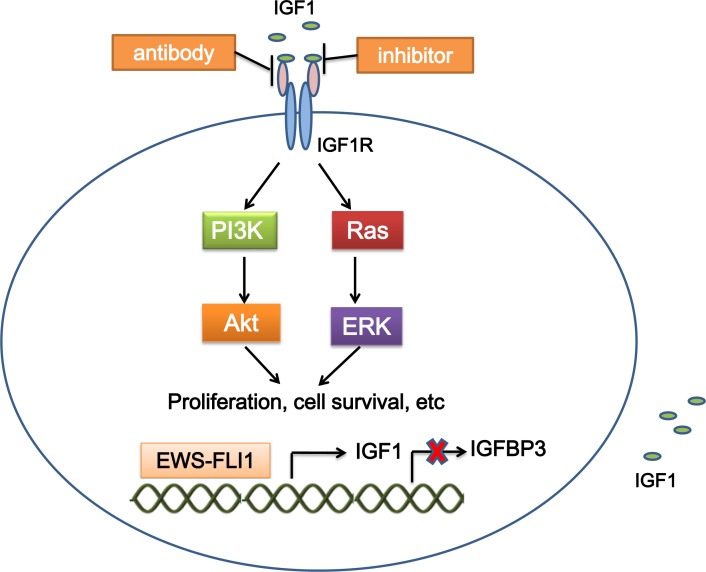
Mechanism of targeting IGF1R in ES EWS-FLI1 binds the promoters of target genes, which increases IGF1 expression, and decreases IGFBP3 expression. IGF1 interacts with IGF1R, and activates the IGF1 pathway, which mediates cellular proliferation, survival, etc. Treatment with IGF1R inhibitors and antibodies suppresses activation of the IGF1 pathway. IGF1, insulin-like growth factor 1; IGFBP3, insulin-like growth factor binding protein 3.

The insulin-like growth factor (IGF) signaling contributes to tumorigenesis [7]. Upon binding to IGF1 (and with less affinity to IGF2), the resulting IGF1R autophosphorylation activates several cancer-related pathways to regulate cell growth and tumorigenesis in a variety of malignancies [8]. IGF1R-mediated loop is constantly present and is a major autocrine circuit in ES [9, 10]. Expression of IGF1R is required for EWS-FLI1-mediated cellular transformation in mouse embryonic fibroblasts (MEFs) and ES cells [11, 12]. EWS-FLI1 activates the IGF1 promoter and induces IGF1 expression in mouse progenitor cells. EWS-FLI1 also binds the promoter of insulin-like growth factor binding protein 3 (IGFBP3) to suppress the expression of IGFBP3 which sequesters circulating IGF1 [13, 14]. These results suggest a crosstalk between the oncogenic function of EWS-FLI1 and the IGF1R signaling (Figure 1).

Interference with the IGF1R pathways in ES cells suppresses growth, increases apoptosis both *in vitro* and *in vivo*, and significantly decreases migration, invasion and metastases [12, 15]. IGF1R targeting is the most studied targeted therapy in ES. Both antibodies and small molecule inhibitors disrupting the IGF1R function are in preclinical and clinical stages of development (Figure 1).

Anti-IGF1R monoclonal antibodies induce responses in a subset of patients with ES. R1507 inhibits the growth of ES cells expressing high levels of IGF2 [16], and exhibits an overall 10% response rate in patients with recurrent or refractory ES [17]. Data from phase I clinical trials suggest that MK-0646 is safe, well tolerated, and significantly inhibits tumor cell proliferation [18]. IMC-A12 (cixutumumab) has broad antitumor activities against ES cells and xenografts [19]. Three (8.6%) out of 35 ES patients had a partial response in a phase I/II trial using IMC-A12 [20]. However, other studies did not show significant benefit from treatment with IMC-A12 for ES patients [21–23]. Treatment with the combination of IMC-A12 and the mTOR inhibitor temsirolimus exhibited a great response in five out of 17 (29%) ES patients [24]. A phase II trial using AMG 479 (ganitumab) in patients with metastatic ES demonstrated a limited antitumor activity [25]. Figitumumab (CP-751,871) showed an antitumor activity in ES cells and xenograft models [26]. Two phase I/II studies using figitumumab in advanced ES resulted in modest responses [27, 28].

In addition to antibodies, the effects of small molecule inhibitors of IGF1R have been investigated in laboratory and preclinical studies. OSI-906 (linsitinib) greatly potentiates the efficacy of trabectedin in ES cells and mouse xenografts. ES patients also showed a preliminary response in a phase I study using OSI-906 [29, 30]. ADW742 inhibits ES cell proliferation and induces apoptosis. Combination of ADW742 and usual chemotherapeutic agents, such as imatinib, vincristine, ordoxorubicin synergistically augmented the effect on ES cells [31]. NVP-AEW541 induces G1 cell cycle block in ES cells [32], and inhibits migration, metastasis, vasculogenicity, and angiogenesis in ES mouse xenografts [33].

#### Other RTKs

C-KIT, a tyrosine kinase receptor, which is expressed in ES, closes an autocrine loop in ES with its ligand stem cell factor (SCF) [34–36]. Platelet-derived growth factor receptor β (PDGFR-β) is also expressed in ES, and regulates cell motility and growth [37–39]. Hence, the KIT/SCF receptor and PDGFR-β may serve as novel targets for molecular-based approaches in ES. Imatinib (Gleevec) is a tyrosine-kinase inhibitor, which selectively blocks tyrosine phosphorylation of KIT and PDGF receptors α and β [40]. Treatment with imatinib inhibits proliferation of ES cells. Administering imatinib orally has an antitumor activity against ES xenografts in mice [41]. One of 7 [42] or one of 24 ES patients [43] had a partial response to Imatinib mesylate in phase II trials, whereas no response was observed in another phase II study [44]. Imatinib increases the sensitivity of ES cells to doxorubicin (DXR) and vincristine (VCR) [40] and improves the outcome of cisplatin or irradiation treatment *in vitro* [45].

Epithelial growth factor receptor (EGFR) promotes cell proliferation and angiogenesis, and EGFR inhibition is used to target tumors. Several attempts have been conducted in ES patients. Andersson et al. reported that EGFR is present in the nuclei as well as localizing to the plasma membrane and cytoplasm in ES cell lines. The cellular proliferation of these cells could be repressed by high doses of gefitinib, a specific inhibitor of EGFR [46]. In another study, gefitinib showed cytotoxic effects in ES SK-NEP-1 cells, whereas little effect on tumor growth was observed in the xenograft models [47]. Pahl et al. found that 2 out of 7 ES cell lines express EGFR, and that anti-EGFR antibody cetuximab enhances the cytolytic activity of natural killer cells toward EGFR-expressing-ES cells [48].

Serum levels of vascular endothelial growth factor (VEGF) are increased in ES patients compared with healthy volunteers, and the serum VEGF levels decrease following neoadjuvant chemotherapy in ES patients [49]. Accordingly, VEGF might serve as a diagnostic and predictive marker of ES. ES cells express VEGF, with an isoform switching from the extracellular matrix-bound 189 isoform to the smaller and more soluble 165 isoform [50]. VEGF-165 expression in the tumor microenvironment contributes to the ES vasculature [51]. VEGF-165 inhibition using small interfering RNA (siRNA) in ES xenografts decreases BM cell migration into the tumor, fewer tumor vessels, and slower tumor growth [52]. Blocking VEGF receptor 2 (VEGFR-2) with a specific antibody significantly reduces tumor growth and tumor vessel density in ES xenografts [53]. Vandetanib, an inhibitor of VEGFR, suppresses tumor cell proliferation [46]. VEGFR2 inhibitor CT-322 inhibits tumor and vessel growth in ES xenograft models [54].

### EWS-FLI1

**Figure 2:**
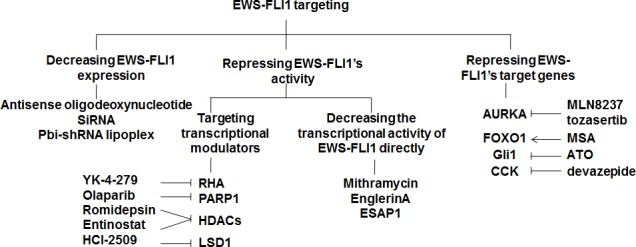
Strategies to target EWS-FLI1 Suppression of the EWS-FLI1 signaling can be achieved by decreasing EWS-FLI1 expression directly using antisense oligodeoxynucleotid, siRNA, or pbi-shRNA lipoplex; repressing the transcriptional activity of EWS-FLI1 by targeting the transcriptional modulators to which EWS-FLI1 binds or the transcriptional activity of EWS-FLI1 itself; or targeting the downstream genes of EWS-FLI1.RHA, RNA helicase A; PARP1, Poly(ADP-ribose) polymerase 1; HDACs, histone deacetylases; LSD1, lysine-specific demethylase 1; AURKA, Aurora kinase A; CCK, Cholecystokinin; MSA, Methylseleninic acid; ATO, Arsenic trioxide.

Transcription factors play an important role in switching genes on and off. In ES, the fusion protein EWS-FLI1, produced by the chromosomal translocation, functions as a transcription factor. EWS-FLI1 induces expression of many factors that promote tumorigenesis, and ES cells die when losing EWS-FLI1. Thus, EWS-FLI1 is a perfect target for treating ES. Targeting EWS-FLI1 can be achieved by decreasing EWS-FLI1 expression through transcription impairment, by decreasing EWS-FLI1 activity through targeting the transcriptional modulators to which EWS-FLI1 binds, or by targeting genes that are deregulated by EWS-FLI1 expression (Figure 2). In contrast to RTK blockade, most studies on targeting the EWS-FLI1 signaling are still in the initial stages of development.

#### Decreasing EWS-FLI1 expression

Either antisense oligodeoxynucleotides [55] or siRNAs [56, 57] could reduce the expression levels of EWS-FLI1, resulting in decreased proliferation of ES cells *in vitro*, and regression of tumors in nude mice. ES xenograft modeling confirmed the dose related safety and tumor response to pbi-shRNA EWS-FLI1 lipoplex [58].

#### Decreasing the activity of EWS-FLI1

##### Targeting transcriptional modulators

**Figure 3:**
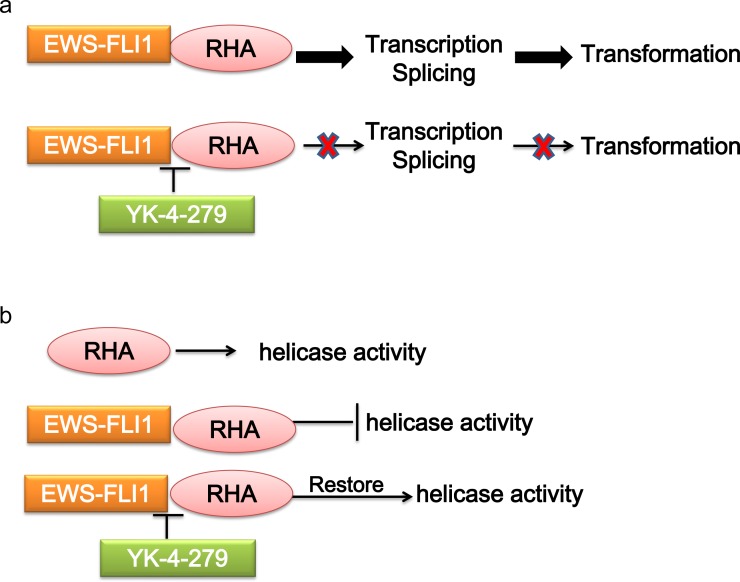
Mechanism of targeting the interaction of EWS-FLI1 and RHA **a**. RHA enhances the transcriptional activity of EWS-FLI1-regulated promoters. The interaction of EWS-FLI1 with RHA affects pre-mRNA processing. **b**. RHA helicase activity is inhibited by EWS-FLI1. Disrupting RHA binding to EWS-FLI1 by YK-4-279 reduces EWS-FLI1 activity, and restores RHA helicase activity. RHA, RNA helicase A.

RNA helicase A (RHA) has multiple functions depending on the specific interactions with different binding partners. In ES, RHA interacts with EWS-FLI1, and functions as a transcriptional modulator. The transcriptional activity of EWS-FLI1 can be enhanced by RHA, which is required by EWS-FLI1 tumorigenic function [59]. The interaction of EWS-FLI1 with RHA affects pre-mRNA processing, resulting in splicing isoforms involved in oncogenesis [60]. On the contrary, RHA helicase activity is also affected by EWS-FLI1, suggesting an important role for the complex interplay between these two proteins in the pathogenesis of ES [61]. There are several approaches to disrupt the RHA binding to EWS-FLI1, thus reducing the EWS-FLI1 activity and tumorigenesis. Small molecule YK-4-279, which has been shown to interrupt the binding of EWS-FLI1 to RHA, induces apoptosis in ES cells and reduces growth in ES xenografts [62–64](Figure 3).

**Figure 4:**
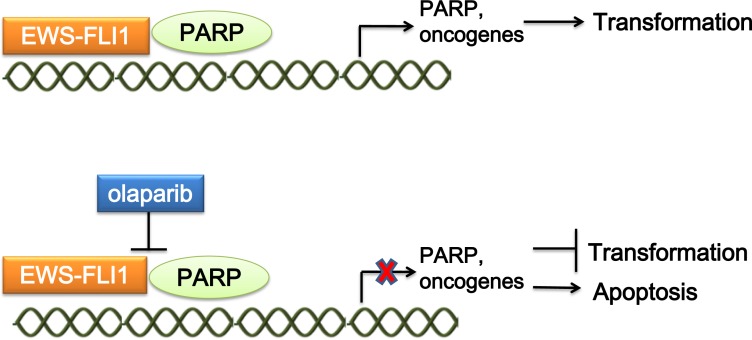
Mechanism of targeting the interaction of EWS-FLI1 and PARP EWS-FLI1 interacts with PARP1, driving PARP1 expression. PARP1 promotes the transcriptional activation by EWS-FLI1. Treatment of ES with the PARP1 inhibitor olaparib could both disrupt the interaction between EWS-FLI1 and PARP1, and impair DNA repair, which represses tumorigenesis. PARP1, Poly(ADP-ribose) polymerase 1.

Poly(ADP-ribose) polymerase 1 (PARP1) is a nuclear enzyme that plays an important role in DNA repair and transcriptional regulation. EWS-FLI1 interacts with PARP1, driving PARP1 expression, which in turn promotes the transcriptional activation by EWS-FLI1. ES cells, primary tumor xenografts, and tumor metastases are highly sensitive to PARP inhibition by olaparib [65]. PARP inhibition in ES disrupts the interaction between EWS-FLI1 and PARP1, and impairs DNA repair [66, 67](Figure 4). Garnett *et al.* identified EWS-FLI1 as a biomarker for PARP inhibition sensitivity in a Cancer Genome Project [68]. Moreover, preclinical studies using ES cell lines showed that the combination of olaparib and radiation amplifies the DNA damage level caused by radiation therapy, synergistically increasing lethal DNA damage [69]. In addition, olaparib can sensitize ES cells to temozolomide-induced apoptosis [70].

Acetylation of histones is associated with chromatin relaxation and transcriptional activation. Histone deacetylases (HDACs) regulate transcription by modifying acetylation of both histones and transcription factors. In ES, EWS-FLI1 expression induces the epigenetic alterations to reprogram cells into the malignancy through activating HDACs. EWS-FLI1 knockdown in ES led to widespread epigenetic changes in promoters, enhancers, and super-enhancers; histone H3K27 acetylation was the most affected mark [71]. An HDAC inhibitor, romidepsin (depsipeptide, FK228), reverses EWS-FLI1 mediated histone deacetylation, decreases *EWS-FLI1* mRNA and protein levels, inhibits cell proliferation, and induces TRAIL-dependent apoptosis of ES cells [72, 73]. The HDAC inhibitor entinostat (MS-27-275) has shown cytotoxicity in ES cells, and a marked regression of established ES xenografts [74].

Histone methylation offers a mechanism to modify chromatin structure and regulate cellular processes, including transcription and genomic stability. Lysine-specific demethylase 1 (LSD1, also known as KDM1A, AOF2 and BHC110), specifically catalyzes oxidative demethylation of mono- and dimethyl-lysine at histone H3 lysines 4 and 9 (H3K4me1/2 and H3K9me1/2) [75]. High LSD1 expression is observed in ES clinical samples, and mechanistic and preclinical studies suggest that LSD1 inhibition globally impairs the function of EWS-ETS proteins. EWS-FLI1 mediated transcriptional repression is facilitated through the direct binding of a NuRD-LSD1 complex [76]. Treatment of ES cells with the LSD1 inhibitor HCI-2509, comprehensively reverses the transcriptional profiles driven by both EWS-FLI and EWS-ERG, and markedly delays tumorigenesis *in vivo* [77].

#### Targeting the transcriptional activity of EWS-FLI1 directly

Mithramycin, which represses EWS-FLI1 activity resulting in a decreased expression of EWS-FLI1 downstream targets, has been used to treat ES patients from 1960s [78, 79]. Mithramycin suppresses growth of ES cells and xenograft tumors, and prolongs survival of mice with ES xenografts [80]. However, the mithramycin toxicity limits its use in ES. Recently, two improved mithramycin analogs, EC-8042 and EC-8105, have shown less toxicity and more efficacy; this could provide opportunities for direct blocking of the EWS-FLI1 activity [81]. EnglerinA (EA), which decreases EWS-FLI1phosphorylation and its DNA binding ability, is an active constituent of the extract from the plant Phyllanthusengleri. EA treatment induces both necrosis and apoptosis in ES cells subsequent to a G2M accumulation of cells in the cell cycle, and inhibits cellular proliferation [82]. EWS-FLI1 binding peptide ESAP1 impairs the transcriptional activity of EWS-FLI1 and blocks the cell cycle progression in ES cells. ES cell growth is reduced by ESAP1, which presents a therapeutic potentiality [83].

#### Targeting the downstream genes of EWS-FLI1

##### Aurora kinase A

The three Aurora kinase (AURK) family members (A, B, and C) are serine/threonine kinases and are key regulators of mitosis as well as diverse signal transduction pathways. Overexpression of Aurora kinase A (AURKA) is associated with tumorigenesis [84]. Wakahara et al. have reported that EWS-FLI1 upregulates the levels of Aurora kinase A and B by directly binding to their promoter regions [85]. The *in vitro* and *in vivo* antitumor activities of an AURKA inhibitor MLN8237 (alisertib) have been reported in ES [86, 87], whereas significant response was not observed in a phase I trial including ES patients [88]. The pan-AURK inhibitor tozasertib selectively reduced the viability of ES cell lines, and inhibited the tumor growth of xenograft models [89].

### FOXO1

Forkhead box O (FOXO) transcription factors are involved in multiple signaling pathways and regulate differentiation, proliferation, tumor suppression, autophagy, and cell death. Microarray results showed that FOX motifs were enriched in the EWS-FLI1-repressed genes, and FOXO1 was transcriptionally repressed by EWS-FLI1 through direct promoter binding. Nuclear FOXO1 overexpression in ES cells impairs cellular proliferation and significantly reduces clonogenicity. Methylseleninic acid (MSA) treatment increased FOXO1 expression in the presence of EWS-FLI1, induced massive cell death and decreased xenograft tumor growth dependent on FOXO1. FOXO1 activation may therefore serve as a promising strategy for targeting ES [90, 91].

#### Gli1

The Gli proteins are transcription factors working as effectors of Hedgehog signaling. Joo et al. showed that primary ES samples expressed high levels of Gli1 [92]. Zwerner et al. reported that NIH3T3 cells expressing *EWS-FLI1* presented malignant phenotype concomitantly with augmented levels of Gli1 [93]. When Gli1 was knocked down, or SUFU, which inhibits Gli1 was overexpressed, the transformed phenotype was decreased, indicating that Gli1 functions downstream of EWS-FLI1 and mediates the transformation induced by EWS-FLI1. In ES cells, EWS-FLI1 knockdown produced a reduction of *Gli1* expression and the transformed phenotype [92, 93]. Chromatin immunoprecipitation (ChIP) studies demonstrated that Gli1 is a direct target of EWS-FLI1 [94]. Arsenic trioxide (ATO) suppresses cell growth by regulating Gli proteins [95]. In ES, ATO is cytotoxic to cells with upregulated Gli1 expression, and decreases xenograft growth [96]. ATO also inhibits migration and invasion of ES cells, indicating a therapeutic potential [97]. In a preliminary study that included ES and metastatic osteosarcoma patients, the combination treatment with ATO and other chemotherapeutic drugs (etoposide and paclitaxel) controlled the tumor growth in 75% of cases [98].

#### Cholecystokinin

Cholecystokinin (CCK) is a peptide hormone, which plays a role in stimulating digestion and modulating intrinsic neuronal excitability. CCK is highly expressed in the majority of ES [99–101]. The prohormone of CCK, proCCK, appears in the supernatant of ES cell lines in culture, and presents a high level in the plasma of ES patients [100]. CCK mRNA levels were upregulated by ectopic expression of EWS-FLI1 [102], and downregulated by EWS-FLI1 knockdown [101]. *CCK* shRNA treatment inhibited cell proliferation *in vitro* and tumor growth *in vivo*, and CCK-rich culture media or exogenous CCK-8 stimulated ES cell proliferation [101, 103]. CCK could bind two G-protein coupled receptors, named CCKAR and CCKBR, to activate numerous pathways to promote cell proliferation. Treatment of ES cell lines with a specific CCKAR antagonist, devazepide, induced apoptosis *in vitro* and significantly reduced the tumor growth in a mouse xenograft model [104]. However, a specific antagonist of the CCKBR (L365 260) did not show any effect on ES cell proliferation or viability [104].

### Mammalian target of rapamycin (mTOR)

mTOR is a central regulator of translation and cell proliferation, which is involved in tumorigenesis of many cancer types [105]. ES expresses mTOR [106], and has an upregulated phosphorylation of mTOR [107]. The effects of mTOR inhibitors on ES have been investigated. Rapamycin efficiently blocked the proliferation of ES cell lines by promoting cell cycle arrest at the G1 phase and decreased the levels of the EWS-FLI1 protein [108]. MLN0128 showed striking antiproliferative effects in ES cells and xenografts [109]. Deforolimus (AP23573; MK-8669) exhibited efficacy in a phase I clinical trial in ES [110]. However, single-agent activity of mTOR inhibitors may be limited by upstream activation of AKT through the negative feedback inhibition, which is mediated in part by IGF1R [111]. Combination of IGF1R and mTOR inhibitors significantly enhanced the antitumor activity in ES cells, xenografts, and ES patients, when compared to either drug alone [22, 24, 112, 113].

### Potential targets

In addition to the targets described above, a number of factors have been reported to regulate the ES tumorigenesis. These factors could be potential targets for the ES targeted therapies in the future. NKX2.2 is an EWS-FLI1-regulated gene that mediates the EWS-FLI1-controlled block of mesenchymal features. NKX2.2 is expressed in ES, and NKX2.2 silencing decreases ES cellular growth and tumor formation in ES xenograft models [114, 115]. DAX1 (NR0B1) is a direct target of EWS-FLI1, and is highly expressed in ES. DAX1 silencing induces growth arrest in ES cells, and severely impairs their growth in semisolid medium and tumor growth in immunodeficient mice [116–118]. PRAS40 is an mRNA target of EWS, and PRAS40 expression is upregulated due to the decline of EWS level in ES. PRAS40 silencing induces the apoptosis of ES cells through regulating the insulin receptor/Akt and mTOR signaling pathways [119, 120]. The chemokine receptors CXCR4 and CXCR7 are expressed in ES, and their expression correlates with poor patient survival [121, 122]. EGR2 is a target gene of EWS-FLI1, and EGR2 knockdown inhibits ES cell proliferation, clonogenicity and spheroidal growth, and induces regression of ES xenografts [123, 124]. CRM1 (XPO1) is a major nuclear exporting protein responsible for trafficking of proteins and RNAs out of the nucleus, and is highly expressed in ES. CRM1 shRNA-mediated silencing or a small-molecule inhibitor KPT-330 treatment in ES cells, dramatically decreases cell growth while inducing apoptosis and cell cycle arrest. CRM1 silencing markedly reduces EWS-FLI1 protein level and upregulates the expression of IGFBP3. Accordingly, attenuation of IGF1-induced activation of the IGF1R/AKT pathway could be a mechanism of CRM1 inhibition for targeting ES [125].

## IMMUNOTHERAPY

Cancer usually escapes or represses the immune responses to enable malignant cells to grow and spread. Cancer immunotherapy aims to establish potent and effective antitumor immune control. Immunotherapies for ES include immune cell-based immunotherapies, cancer vaccines, and monoclonal antibodies.

### T cell-based immunotherapy

#### T cell priming by ES associated antigens

T cells usually target only cells expressing antigenic sequences presented to the T cell receptor *via* MHC. However, advanced ES patients fail to express MHC molecules and have defective antigen processing. The abnormal tumor-immune cell interactions in ES may prevent antitumor immunity [126]. Therefore, improvement of antigen processing and interactions between ES and T cells has been a focus of ES immunotherapies.

**Figure 5:**
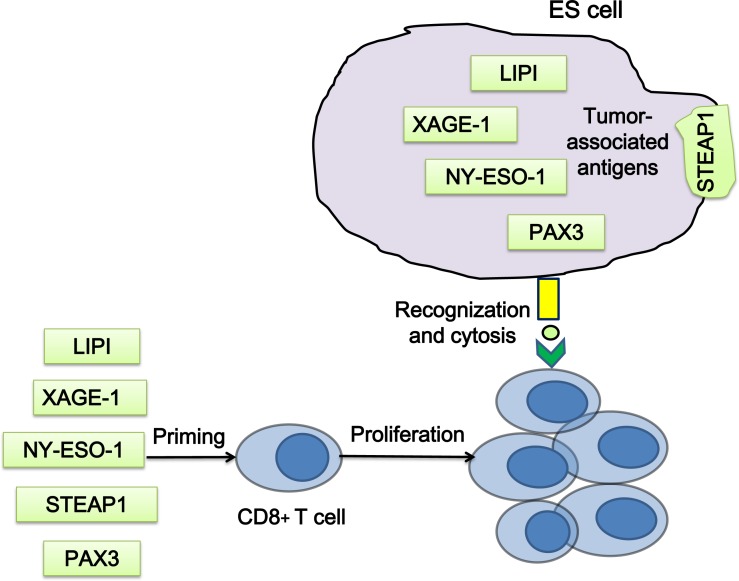
Strategies of T cell priming by ES associated antigens T cells, which are primed by the peptides of the tumor associated antigens *in vitro*, could recognize and kill ES cells when transferred into patients. LIPI, phospholipase A1 beta; XAGE1, X antigen family member 1; NY-ESO-1, New York esophageal squamous cell carcinoma 1.

Since the tumor-specific EWS-FLI1 is an ideal tumor antigen, it has served as an important immune target. However, peptides derived from the fusion protein are insufficient to induce CD8^+^ cytolytic T-cell responses [127, 128]. Due to the absence or low expression in adult somatic tissues, cancer/ testis antigens are considered as potential targets for T cell priming in ES, which include phosphatidic acid specific membrane-associated phospholipase A1 beta (lipase I, LIPI), X antigen family member 1 (XAGE1) and New York esophageal squamous cell carcinoma 1 (NY-ESO-1, also known as CTAG1). LIPI is highly expressed in ES, and has a high tumor specificity for ES [129, 130]. Cytotoxic T lymphocytes (CTLs) specific for LIPI-derived peptides LDYTDAKFV and NLLKHGASL could lyse HLA-A2 positive ES cells *in vitro* [131]. XAGE1 is strongly expressed only in normal testis and ES [131–133]. The NY-ESO-1 expression in ES has been reported [134], and T cells showed a response to the MAGE-A1, MAGE-3 and NY-ESO-1 peptide mix [135](Figure 5).

STEAP1 is a transmembrane protein in epithelial cells, the expression of which in normal tissues is restricted to prostate and bladder [136]. STEAP1 is highly expressed in ES [137, 138], and its expression correlates with the EWS-ETS fusion protein [139]. Thus, STEAP1 is a suitable antigen for T cell priming and chimeric antigen receptor (CAR) T cells targeting. However, a recent study has reported a failure to induce T-cell response in ES by STEAP1 and XAGE1 primed T cells [140]. The transcription factor PAX3 is expressed in ES and functions as a general tumor associated antigen. Two peptides, PAX3-282 (QLMAFNHLI) and its modified version PAX3-282.9V (QLMAFNHLV), were capable to induce antigen-specific CTLs, which were found to be able to lyse ES cells expressing PAX3 [141](Figure 5).

#### CAR T cells

CAR T cells are genetically engineered cells that produce special receptors on their surface to allow T cells to recognize a specific antigen on tumor cells. IGF1R and ROR1 CAR T cells showed cytotoxic responses against sarcoma cells including ES [142].

### Natural killer (NK) cell-based immunotherapy

Besides antigen-specific CTLs, NK cells are highly cytotoxic immune cells that can kill tumor cells *via* release of cytotoxic granules inducing tumor apoptosis. Unlike T cells, NK cells interact with tumor cells independently of antigen presentation, and thus bypass the major mechanisms of immune escape. Low expression of MHC class I [126, 143] and high expression of NK cell receptor activators in ES suggest a potential role of NK cells in immunotherapy for ES [144]. ES cells and xenografts are exquisitely sensitive to NK cells due to the expression of NK cell receptors, NKG2D and DNAM-1 [144, 145]. Although chemotherapy-resistant ES exhibited reduced susceptibility to resting NK cells, it could be restored by interleukin-15-mediated activation of NK cells. Pretreatment with an HDAC inhibitor induced NKG2D-ligand expression [146], suggesting yet another mechanism of HDAC inhibition function in ES.

### Cancer vaccines

Oncolytic viruses (OV) can selectively infect and kill tumor cells, while sparing normal tissues. Phase I/II clinical trials using OV in carcinoma patients demonstrated remarkable safety profiles and notable clinical effects. A systemic delivery of vesicular stomatitis virus (VSVΔM51) resulted in a significant decrease of tumor burden in ES bearing mice, and tumor-specific killing of resistant ES cells [147].

Dendritic cells (DCs) are the most potent professional antigen-presenting cells of the immune system. DCs present internalized antigens derived from exogenous sources, not only on MHC class II molecules, but also on MHC class I molecules, to CD8^+^ T cells. In DCs stimulated by EWS-FLI1, the specific CTL response against ES cells or xenografts was induced successfully [148, 149]. Combination of autologous lymphocytes, tumor lysate /keyhole limpet hemocyanin-pulsed DC vaccinations and recombinant human IL7, following standard antineoplastic therapy, resulted in higher survival rates, and lower recurring rates in ES patients, compared with patients receiving standard antineoplastic therapy [150]. However, vaccination with DCs pulsed by peptides derived from the breakpoint region of EWS-FLI1 did not alter the clinical outcome for patients with recurrent ES [151].

### Monoclonal antibodies

CD99 (also called MIC2) is a cell surface transmembrane glycoprotein highly expressed in ES [152–154]. CD99 inhibits neural differentiation, and induces cell proliferation and tumor growth in ES through controlling the MAPK, Notch-NF-κB or p53 pathways [155–157]. Engagement of CD99 using anti-CD99 antibody induces massive apoptosis of ES cells through caspase-independent mechanisms and reduces their malignant potential [158–160]. In athymic nude mice, the combined treatments with anti-CD99 0662 monoclonal antibody and doxorubicin (DXR) were remarkably effective against both local growth and metastases of ES xenografts, and the survival rate of mice was also increased [161].

## CONCLUSION AND FUTURE PERSPECTIVES

Development of the anti-ES treatments has been delayed due to the rarity of the disease. However, effective treatments are needed urgently because of the low survival rates of ES patients. With the increasing focus on rare diseases and the global cooperation, the novel approaches including targeted therapies and immunotherapies have been recently investigated in ES. Despite the conceptual promise, the effect of most strategies is still modest, especially since resistance has been a problem, and has impaired the impact on clinical outcomes. Fortunately, combination therapies may enhance the efficacy and decrease the resistance. RTK inhibitors have shown synergistic effects with chemotherapeutic agents [40, 45]. mTOR inhibitors have been more effective in combination with other agents, such as IGF1R inhibitors [22, 24, 112, 113]. Since combination therapies often decrease the drug resistance, future studies should explore their use in the ES treatment.
